# Secondary monitor unit calculations for VMAT using parallelized Monte Carlo simulations

**DOI:** 10.1002/acm2.12605

**Published:** 2019-05-24

**Authors:** Stephen Bhagroo, Samuel B. French, Joshua A. Mathews, Daryl P. Nazareth

**Affiliations:** ^1^ Department of Radiation Medicine Roswell Park Comprehensive Cancer Center Buffalo NY USA; ^2^ Department of Radiation Oncology Piedmont Healthcare Atlanta GA USA; ^3^ Medical Physics Program University at Buffalo (SUNY) Buffalo NY USA

**Keywords:** algorithms, independent verification, monitor units, Monte Carlo methods, secondary check, simulation, VMAT

## Abstract

We have developed a fast and accurate in‐house Monte Carlo (MC) secondary monitor unit (MU) check method, based on the EGSnrc system, for independent verification of volumetric modulated arc therapy (VMAT) treatment planning system dose calculations, in accordance with TG‐114 recommendations. For a VMAT treatment plan created for a Varian Trilogy linac, DICOM information was exported from Eclipse. An open‐source platform was used to generate input files for dose calculations using the EGSnrc framework. The full VMAT plan simulation employed 10^7^ histories, and was parallelized to run on a computer cluster. The resulting *3d*dose matrices were converted to the DICOM format using CERR and imported into Eclipse. The method was evaluated using 35 clinical VMAT plans of various treatment sites. For each plan, the doses calculated with the MC approach at four three‐dimensional reference points were compared to the corresponding Eclipse calculations, as well as calculations performed using the clinical software package, MUCheck. Each MC arc simulation of 10^7^ particles required 13–25 min of total time, including processing and calculation. The average discrepancies in calculated dose values between the MC method and Eclipse were 2.03% (compared to 3.43% for MUCheck) for prostate cases, 2.45% (3.22% for MUCheck) for head and neck cases, 1.7% (5.51% for MUCheck) for brain cases, and 2.84% (5.64% for MUCheck) for miscellaneous cases. Of 276 comparisons, 201 showed greater agreement between the treatment planning system and MC vs MUCheck. The largest discrepancies between MC and MUCheck were found in regions of high dose gradients and heterogeneous densities. By parallelizing the calculations, point‐dose accuracies of 2‐7%, sufficient for clinical secondary checks, can be achieved in a reasonable amount of time. As computer clusters and/or cloud computing become more widespread, this method will be useful in most clinical setups.

## 1 INTRODUCTION

Treating patients with therapeutic radiation is a dynamic process with little room for error and potentially fatal consequences in cases of misadministration. As such, the process is accompanied by chart reviews and independent monitor unit (MU) calculations.^1^ In the past, separate independent hand calculations were the primary method of verification. However, sophisticated linac hardware, coupled with advanced treatment planning algorithms, has made possible complex treatment delivery modalities. This is especially true in the case of volumetric modulated arc therapy (VMAT), which involves the computation and optimization of hundreds of multivariable control points across multiple treatment arcs.^2^ Given the stringent demands on a physicist's time at a radiotherapy practice, sophisticated computer algorithms should be employed as part of the secondary MU verification process, as per TG‐114 guidelines.^1^

In addition, secondary MU verification also serves a diagnostic purpose. Standard treatment planning system (TPS) commissioning tests are not capable of evaluating system responses over the entire range of possible treatment scenarios that may be designed. Any bugs in the system code or erroneous module performances may be discovered through the secondary MU verification process.^3^, ^4^ As such, it is important that the verification system works independently of the hospital's commissioned TPS software. Our center uses Varian's Eclipse Ver. 13 Treatment Planning System (Varian Medical Systems, Palo Alto, CA) for all external‐beam patient plans.

Monte Carlo (MC) algorithms provide the most accurate models of radiation transport by accounting for all relevant physical processes involved in each particle's interaction history.^5^ The MC calculations also do not require approximations of the source to surface distance (SSD) or calculation depth for each control point. As a result of this comprehensive approach, a VMAT MC calculation requires significant computing resources to produce accurate results in a reasonable amount of time, and is not commonly employed for VMAT secondary MU checks. However, with cloud and cluster computing becoming more widespread, clinical use of MC calculations may be feasible. For this work, the computational resources of an academic supercomputer cluster were used.

Commercial software used for performing secondary MU checks vary in sophistication and dose calculation algorithms, but most use some form of modified Clarkson integration, as is the case for RadCalc (Lifeline Software Inc., Austin, TX), Diamond (PTW, Freiburg, Germany), and IMSure (Standard Imaging Inc., Middleton, WI).^6^ One study^6^ evaluated the performance of these software packages in comparison to the Pinnacle3 TPS (Philips Medical Systems, Bothell WA). The study found that for 59 VMAT arcs, IMSure, and Diamond produced the most outlying results, with the greatest variance in accuracies compared to the TPS, while RadCalc was found to be the most consistent and most accurate of these software packages.

Heterogeneity corrections are often a concern when using VMAT secondary check software. Most secondary check software employs simplistic heterogeneity corrections using density approximations and incorporating tissue only in the immediate area of the point of interest.^7^, ^8^, ^9^, ^10^ This can be problematic, as local energy absorption and lateral scatter can change significantly across different types of tissue. In addition, secondary check software for VMAT plans usually only consider dose to a single point. While this is recommended in the TG‐114 report, a more sophisticated comparison using the entire dose matrix of the treatment volume may be useful for the relatively complex treatment plans designed for VMAT (see Section 2.2).

MUCheck software with the VMAT Module (Oncology Data Systems, Inc., Oklahoma City, OK) is currently used as the independent dose calculation method for VMAT plan secondary verifications at our institute. The dose calculation method utilized in MUCheck is comparable to other similar secondary check software, and is much simpler than that of Eclipse TPS and MC algorithms.^7^, ^11^ Dose is calculated to a single point within the phantom by first tracing a ray to the x‐ray source. Voxel density and any interfaces between tissue types are only accounted for along the ray; any adjacent inhomogeneities are not included in the calculation. Dose contributions from each leaf pair in a control point are considered in the calculation, using a method similar to the modified Clarkson integration, but using symmetric rectangles based on the four corners of each leaf pair, instead of annular integration. The boxes are then mathematically subtracted and weighted based on their off‐axis ratios (OARs), measured from their centers.

## 2 MATERIALS AND METHODS

**Figure 1:**
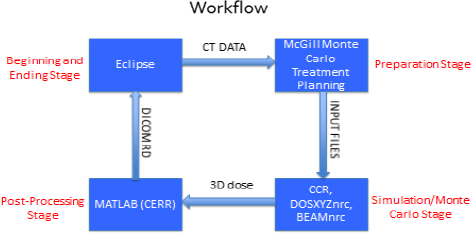
Schematic of the overall workflow

Running an MC calculation as a secondary MU check protocol is a multistep process involving the creation and transfer of a large number of files. The following is a description of the process for running a calculation, processing the output data and analyzing the results. A schematic of the overall workflow is depicted in Fig. 1. Using BEAMnrc, a utility in EGSnrc which allows for building a medical accelerator, a Varian Trilogy linac was modeled. Every component in the linac head was modeled based on the dimensions from manufacturer specifications, and assigned a specific material and corresponding density. The machine was configured for 6 MV photon treatments only. Similarly, DOSXYZnrc, an EGSnrc utility used to model dose deposition in a voxelated computed tomography (CT) phantom, was used for calculating patient dose. McGill Monte Carlo Treatment Planning System (McGill University, Montreal, Quebec) provides GUI‐based functionality for changing different parameters of the accelerator (mlc shapes and jaw position generation for specific patient plans) and was used to generate patient‐specific simulation files.

### 2.1 Calibration factor

The EGSnrc calculation produces a *3ddose matrix*, which assigns a floating‐point value to each voxel in the phantom, in units of Gy/particle. In order to convert these values to Gy, a *calibration factor* must be determined. The calibration factor was obtained by performing an MC calculation of a static plan under standard conditions (10 × 10 field size, 100 cm SSD, 40 × 40 × 40 cm water block phantom) in DOSXYZnrc. An identical plan was created in Eclipse using 100 MUs. The energy used in both plans was 6 MV. In order to obtain the most accurate results possible within a reasonable time frame, the MC calculation was set to simulate 10^10^ particles.

**Figure 2:**
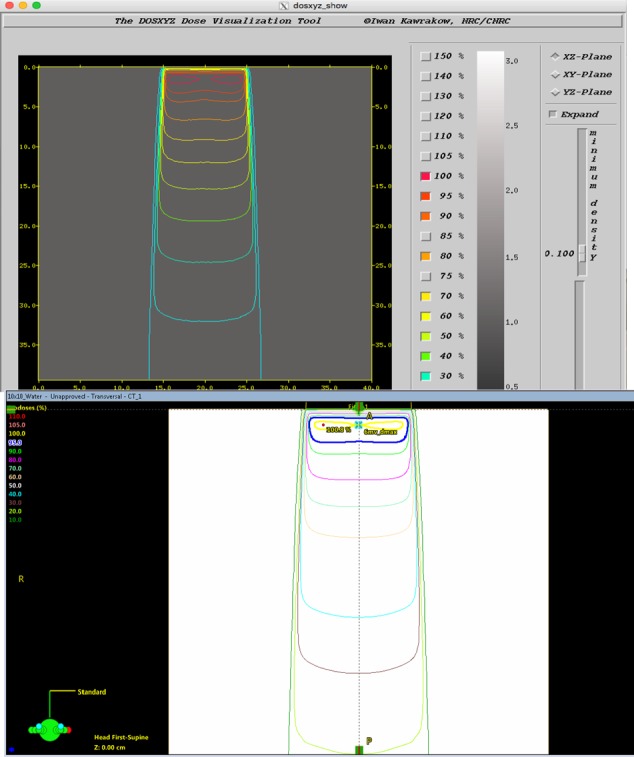
(a and b) Top: a view the 10 × 10 isodose profile obtained in DOSXYZ. Bottom: a view of the 10 × 10 isodose profile obtained in Eclipse.

The results were compared to the Eclipse plan, as shown in Fig. 2. The doses at d_max_ on the central axes in the two plans were used to determine the MC calibration factor, F_cal_, as follows.

$$\text{Eclipse}\;d_{\max}\;\text{Dose}:100\%\;of\;1.004438\;\text{cGy/MU}=0.0100444\;\text{Gy/MU}$$

$$\text{Monte Carlo}\;\text{CAX}\;\left(10^{10}\text{particles}\right):\;1.43953\times 10^{-16}\;\text{Gy/Particle}$$

$$F_{\mathrm{Cal}}=\frac{0.0100444\;\text{Gy/MU}}{1.43953\times 10^{-16}/\text{Particle}}=6.98\times 10^{13}\;\frac{\text{Particle}}{\text{MU}}$$

This method of obtaining absolute dose values from MC calculations is similar to the formalism described by Francescon.^12^ Once F_cal_ is determined, it can be applied to any MC calculation employing the same linac model. All MC‐calculated dose matrices were multiplied by both F_cal_ and the MU number specified in Eclipse for a given VMAT arc, in order to obtain absolute dose values for each case.

### 2.2 Generation of files for simulation

For each plan, all DICOM files, including CT images, were exported from Eclipse to a folder in the main McGill Monte Carlo Treatment Planning (MMCTP) system directory, where the MMCTP data import routines were run. Using RTOG software, all unique patient identifiers and tags were removed from exported Eclipse files. The program prompts the user to input a label comprised of an institute abbreviation, a plan type, and case number, which is applied to all files associated with the VMAT plan. Each patient's label is systematically logged and matched with the corresponding patient ID, provided by Eclipse. Plan parameters were specified using the MMCTP interface, which then generated the input files for the DOSXYZnrc and BEAMnrc routines.

Four different material types with lower and upper density ranges were defined and scaled according to the Hounsfield Unit (HU) information in the CT data. These density data were used by MMCTP to create a voxelated phantom with the DOSXYZnrc routine. Table 1 lists the phantom tissue types in each phantom and their densities based on HUs. MMCTP allows for adjustment of the type and number of materials used, as well as scaling of density to CT data.

**Table 1:** Phantom material densities based on Hounsfield unit (HU)

Material	HU low	HU high	Density low (g/cm^3^)	Density high (g/cm^3^)
Air	−1050	−985	0.001	0.001
Lung	−985	−810	0.001	0.24
Tissue	−810	85	0.24	1.19
Bone	85	4000	1.19	3.75

### 2.3 Running and importing a simulation

An academic supercomputer facility was used to run all MC calculations. All necessary file transfers were performed using open‐source file transfer protocol (FTP) and secure shell (SSH) clients. In order to optimize the MC calculation efficiency, each calculation was split into multiple jobs, by creating multiple separate input files. The number of jobs depends on the computing resources available; for this study, the number ranged from 100 to 1000. For each VMAT calculation, the total number of simulated histories was in the range of 10^6^ to 10^8^. The batch computing scheduling and resource management system used was the Simple Linux Utility for Resource Management (SLURM). The output of each EGS job is a partial dose calculation, or *pardose* file. After all jobs are complete, a script is run to sum the pardose files into a single *3ddose* file. The 3ddose file can then be inserted into the original DICOM‐RD file using Matlab (MathWorks Inc., Natick, MA) routines, for importing into Eclipse.

In Eclipse, a new patient plan with an empty dose matrix was created for each patient arc. Once the MC absolute dose matrices were calculated using F_cal_, as described above, they were imported into each empty plan. No further normalization was used for any MC or Eclipse plans during the comparison. Thus, the absolute dose at each point in the MC plans and corresponding Eclipse plans could be compared.

### 2.4 Evaluations

All treatment plan data used for evaluations were acquired from our department's Eclipse TPS patient database. A set of 35 single and multi‐arc clinical VMAT treatment plans were assembled, comprised of ten prostate, ten head and neck, ten brain cases, and five miscellaneous cases.

Four three‐dimensional reference points were identified in each plan, each located 1 cm from the isocenter, in the superior/inferior and lateral directions. For selected plans, reference points were also added in heterogeneous and steep dose fall off regions. For each reference point, the percent dose difference was determined between the Eclipse and MC dose calculations. For comparison, the percent MU difference was also determined between the Eclipse and MU Check calculations. In addition, the MC calculation time required for each patient case was recorded.

## 3 RESULTS

### 3.1 Disagreement at a single reference point

**Figure 3:**
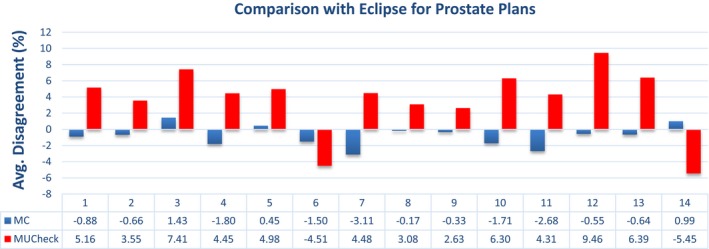
Disagreement in Eclipse vs Monte Carlo calculations (blue) and Eclipse vs MUCheck (red) for prostate plans, evaluated at one reference point. There were a total of 14 arcs and 10 plans for the prostate cases.

**Figure 4:**
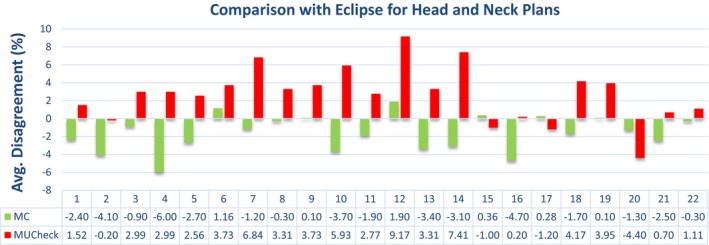
Disagreement in Eclipse vs Monte Carlo calculations (green) and Eclipse vs MUCheck (red) for head and neck plans, evaluated at one reference point, for head and neck plans. There were a total of 22 arcs and 10 plans for the head and neck cases.

**Figure 5:**
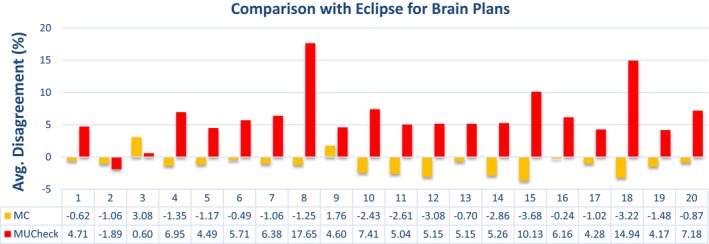
Disagreement in Eclipse vs Monte Carlo calculations (yellow) and Eclipse vs MUCheck (red) for brain plans, evaluated at one reference point. There were a total of 20 arcs and 10 plans for the brain cases.

**Figure 6:**
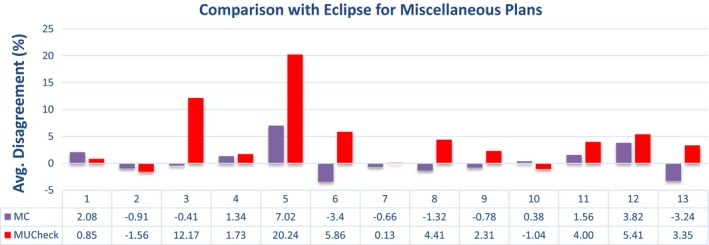
Disagreement in Eclipse vs Monte Carlo calculations (purple) and Eclipse vs MUCheck (red) for miscellaneous plans, evaluated at a single reference point. There were a total of 13 arcs and 5 plans for the miscellaneous cases.

The data presented in Figs. 3, 4, 5, 6 show a sampling of the disagreement between the MC and Eclipse absolute doses to a reference point, as well as the disagreement between MUCheck and Eclipse absolute doses to the same reference point. Each dose comparison is made for one VMAT arc. It is clear that the MC calculations generally show better agreement with Eclipse.

**Figure 7:**
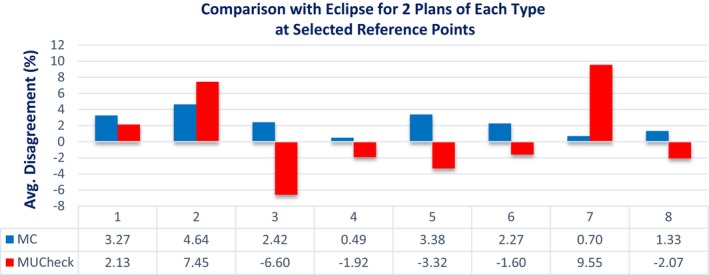
Disagreement in Eclipse vs Monte Carlo calculations (blue) and Eclipse vs MUCheck (red) for 8 plans (two each of prostate, brain, head and neck, and miscellaneous plans,) at a reference point placed in a region of heterogeneity or steep dose gradient.

Figure 7 shows the disagreement between MC and Eclipse as well as MUCheck and Eclipse absolute doses for reference points specific to each plan. These reference points were chosen to be in heterogeneities or dose gradients, located two to three centimeters from the isocenter. MC showed better agreement with Eclipse in most cases. For the lung case (number 7), MUCheck showed particularly high disagreement with Eclipse.

**Figure 8:**
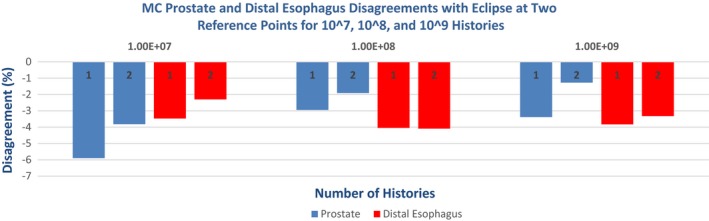
Change in percent disagreement with Eclipse at two separate reference points for the same Monte Carlo calculated prostate and distal esophagus plans as number of histories increases.

Figure 8 shows how the disagreement with Eclipse for two MC‐calculated plans and two reference points varies with the number of histories used. In these results, all MC calculations resulted in lower doses than those of Eclipse. The maximum absolute disagreement decreases from 5.9% (10^7^ histories) to 3.8% (10^9^ histories). Further reduction in disagreement values may not be possible due to the limitations of Eclipse's AAA algorithm (see Section 2.2).

**Figure 9:**
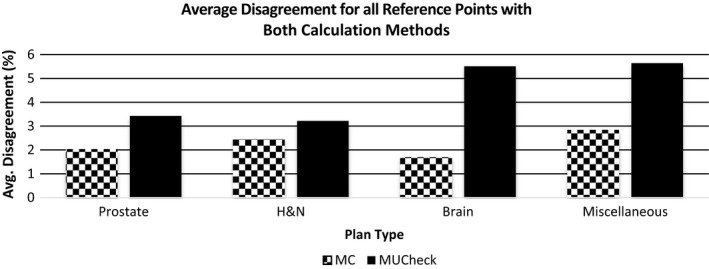
Average absolute disagreement in Eclipse vs Monte Carlo calculations (gray) and Eclipse vs MUCheck (black) over four reference points, separated by plan type.

Figure 9 shows the average of the absolute value of disagreement over all four reference points for the Eclipse vs MC calculations and Eclipse vs MUCheck, grouped by plan type. The values for MC are significantly lower (1.7%–2.8%) than those of MUCheck (3.2%–5.6%). Although the best agreement for Eclipse vs MC occurs for the brain cases, the Eclipse vs MUCheck results for the brain cases are among the worst in agreement (see Section 2.2).

### 3.2 Calculated dose visualization

**Figure 10:**
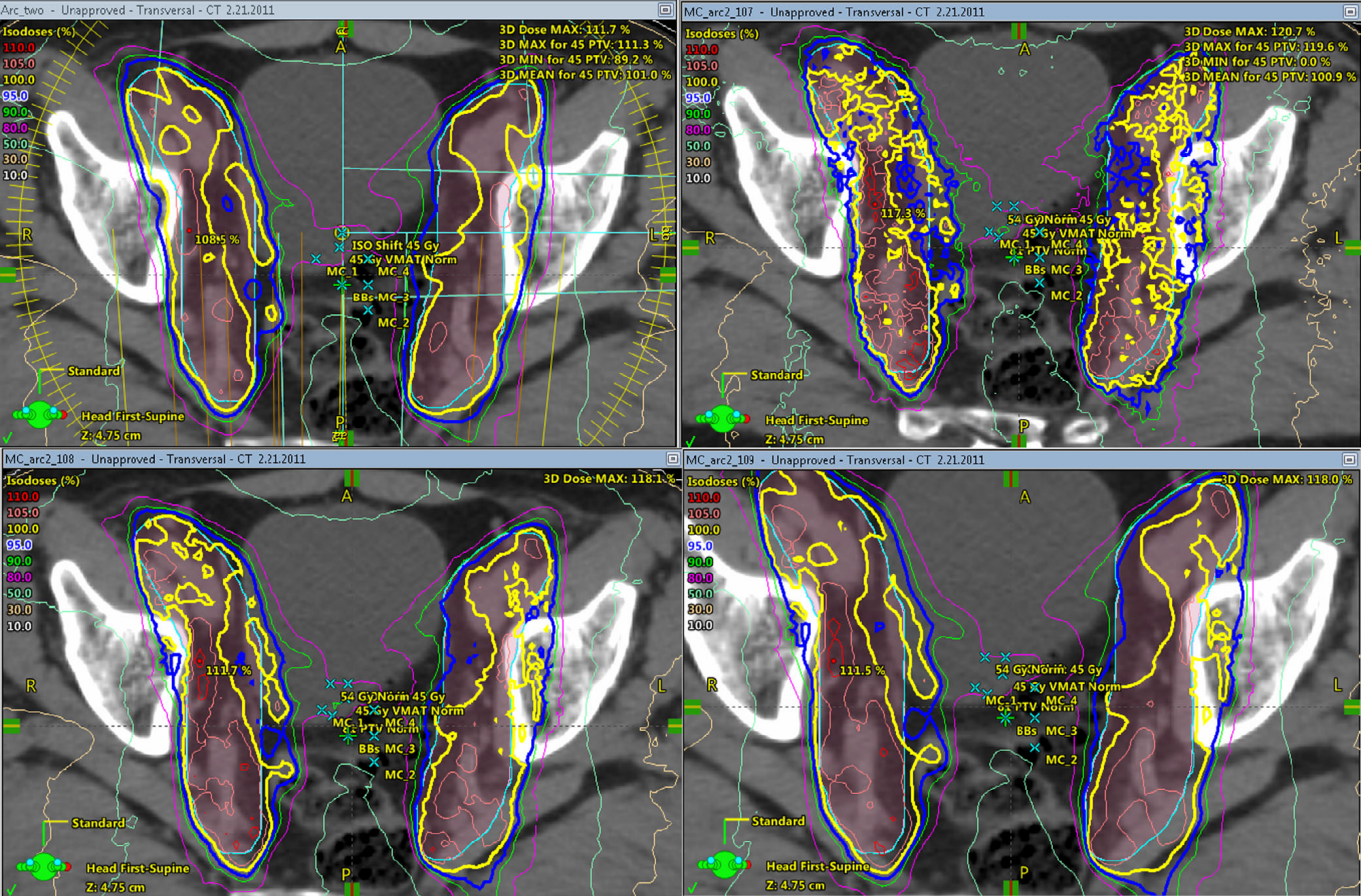
Isodose lines in transverse view for one prostate case. Top left: eclipse; Top right: Monte Carlo (MC) with 10^7^ histories; Bottom left: MC with 10^8^ histories; Bottom right: MC with 10^9^ histories.

As a visual comparison of the MC and Eclipse dose calculations, Fig. 10 presents a transverse view of isodose lines for one of the evaluated prostate cases. Three MC calculations are shown, corresponding to 10^7^, 10^8^, and 10^9^ histories. The reduction in noise of the MC plans is evident as the number of histories is increased.

### 3.3 Job execution times

**Figure 11:**
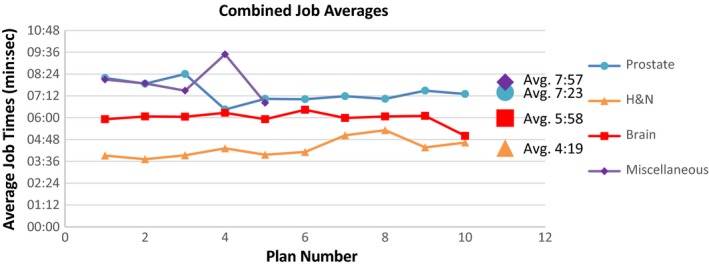
Average job time for each patient and plan, and overall average per group.

Figure 11 presents the average MC calculation times for 100 jobs for one arc of each patient plan, separated by plan type. The average of the averages for each group is included. The prostate and miscellaneous cases required the highest run times, probably due to their large calculation volumes.

Generation of input files from imported DICOM files took an average of 10 min per plan. The estimated range of overall calculation time, from generation of input files to job completion, was about 13–25 min.

## 4 DISCUSSION

The results obtained from this work indicate that employing a Monte Carlo‐based approach for VMAT secondary checks provides useful and accurate results, which match or outperform commercial secondary check software. The average agreement with Eclipse's calculations for all groups was below 3%, which exceeds even the most stringent secondary check accuracy thresholds.^13^ There were 15 out of 276 cases that exceeded 5% agreement. In general, these were the results of calculations performed for small fields or where inhomogeneities were present. In some cases, using more particles such as 10^8^ or 10^9^ at the same reference point did lower percent deviations for that point, though the agreement with Eclipse actually decreased for some reference points using 10^9^ particles. This is potentially a result of the MC algorithm modeling the physics of radiation transport more accurately than Eclipse, which uses the Analytical Anisotropic Algorithm (AAA) to calculate dose.^9^ The limitations of type‐b, kernel‐based algorithms such as AAA are well known.^9^, ^14^, ^15^ Future work will involve dose calculated in Eclipse by a more sophisticated, type‐c algorithm, such as Acuros XB.^16^

Through the application of parallel processing, calculation times for each secondary check were reasonably low, with all jobs completing in <10 min. Head and neck plans had the lowest average job calculation times [4:19] (in min:s), followed by brain plans [5:58], prostate plans [7:23], and miscellaneous cases [7:57]. The prostate and abdomen plans involve relatively large calculation volumes, which can explain longer calculation times. The thoracic region (especially the lungs) is relatively inhomogeneous, and as a result, calculations are more complex and time consuming.

The VMAT simulations carried out in this study were performed using an academic cluster. For comparison, a benchmark simulation was carried out on a 16 core desktop featuring a previous generation Sandy Bridge processor, and another 16 core desktop featuring a current generation Skylake processor, resulting in total calculation times of about 65 and 19 min, respectively. While the former is considerably longer than the calculation times obtained in this study, it still may be made acceptable by modifying the typical clinical workflow. Employing local parallel processing using a few 16 or 32 core clinical desktops, especially those featuring the latest generation technology, could result in much more practical calculation times.

An MC simulation also provides a complete dose matrix calculation over the full patient anatomy of interest, which can be imported into the TPS. As a result, more sophisticated analysis can be conducted than with typical secondary MU check methods. For instance, prescription dose coverage of the entire target volume can be compared between the MC data and Eclipse data, and gamma index and distance to agreement (DTA) analysis can be used to obtain passing rates, similar to IMRT quality assurance analysis. Isodose lines and dose‐volume histograms may also be compared. This can be especially useful with plans that involve small volumes or tumors in difficult to treat areas. It should be noted that, at our center, physical quality assurance measurements are performed for every patient before VMAT treatment is administered. The secondary monitor unit check method presented here does not supersede this QA, but rather supplements it.

Since most MC calculations using 10^7^ histories resulted in agreements within 5%, this can be the default number of histories used. The longest overall calculation time was 9:29 min. There are several possible ways of reducing this time. One method proposed by French et al.^17^ suggests that compiler optimization, as well as a strategic choice of random number generators, can result in a speedup of VMAT MC calculations of 23%. Another possibility involves an effective combination of parallelization of computation and selection of the number of histories. In addition, cloud‐based MC verifications are now becoming quite feasible and can be cost‐effective,^18^ especially when savings are factored in from the use of the open‐source and freely available EGSnrc software. The fractional error in an MC calculation is inversely proportional to the square root of the number of histories. This implies that improving the accuracy by a factor of 10 would require 100 times more histories. The best approach is therefore to select a value that represents a balance between the desired level of accuracy and the computing time/resources available.

Monte Carlo simulations, while robust and accurate in calculation methods and ability to model physical phenomena, do involve trade‐offs between speed and accuracy, as mentioned above. Accuracy is determined by the particle count used for a simulation, which in turn affects calculation speed. For clinics not equipped with the necessary hardware, calculation time may be a significant burden when treatment plan physics checks are performed. Additionally, the EGS/BEAMnrc platform is more complex and has a steeper learning curve than that of user‐friendly commercial software. These aspects of a Monte Carlo‐based approach should be properly considered before implementation.

Out of 276 point‐dose comparisons performed in this study, 201 (73%) resulted in greater agreement between MC doses and TPS doses than between MUCheck and TPS doses. The highest deviation for an MC calculation was −7.95%; the highest deviation for a MUCheck calculation was 83.16%. The average percentage deviations for the prostate, head and neck, brain, and miscellaneous cases were lower for MC calculations than MUCheck calculations [2.3% vs 4.45%]. It should be noted that there are considerable limitations in the MUCheck calculation algorithm, as stated in the MUCheck VMAT module manual, and summarized above. Additionally, points chosen that are typically located on a leaf pair boundary, such as isocenter, will result in large inaccuracies with MUCheck, which may explain some of the values obtained in this study. A future investigation will also involve a comparison of MC‐calculated dose with MUCheck calculated dose in SBRT/flattening‐filter free cases, for which MUCheck has shown considerable disagreement with Eclipse, in our clinical experience.

Agreements between MUCheck and Eclipse for brain cases were among the worst presented in this study. Though the density and composition of brain matter is relatively homogeneous and should not present any significant dose calculation challenges, MUCheck assumes that the patient surface at the area of beam incidence is flat. This approximation may lead to large dose calculation errors, but is not an issue for the MC method.

## 5 CONCLUSION

The aim of this project was to develop an EGSnrc‐based Monte Carlo calculation system as a viable secondary MU check method for VMAT plans, and show that the calculation times would make the system clinically feasible to implement. This method compares favorably with commercial secondary check software. The average disagreement between the MC and TPS calculations were between 1.70% and 2.84%, which are well within those recommended by the TG‐114 protocol. The average calculation time ranged from 4:19 to 7:57 min.

VMAT plans are among the most complex radiotherapy treatments to design and deliver. As a result, a sophisticated secondary check algorithm should be employed to ensure the integrity of each treatment plan. The EGSnrc‐based MC approach used in this study is significantly more accurate than algorithms commonly used in third‐party commercial software, which often includes simplifications of geometry and particle interactions. Overall, given the constant progression of computing power and the integration and expansion of cloud computing, this open‐source approach could become a widely available tool for radiation medicine centers that employ VMAT treatment.

## CONFLICTS OF INTEREST

No conflicts of interest.
